# Supplementary material for: Impact of Smoking Status on Lung Cancer Characteristics and Mortality Rates between Screened and Non-Screened Lung Cancer Cohorts: Real-World Knowledge Translation and Education
Source: J Pers Med. 2022 Jan 2;12(1):26. doi: 10.3390/jpm12010026 (PMC8780024; doi:10.3390/jpm12010026)
Supplement: Supplementary file 1 [file jpm-12-00026-s001.zip › jpm-1448189-supplementary.pdf]

**Supplement Table S1.** Clinical characteristics of 1651 non-smokers with lung cancer according to screening status.

| | **Screened (n = 81)** | **Non-Screened (n = 1570)** | ***p*-Value** |
|---|---|---|---|
| Mean age at diagnosis, years (mean, SD) | 59.41 ± 7.41 | 66.36 ± 12.78 | <0.001 |
| Median age at diagnosis, years (range) | 66 (42-77) | 66 (40-99) | |
| Gender (n, %) | | | 0.159 |
| Male | 24 (29.6%) | 589 (37.5%) | |
| Female | 57 (70.4%) | 981 (62.5%) | |
| Alcohol drinking consumption | 4 (4.9%) | 39 (2.5%) | 0.156 |
| Betel nut consumption | 0 (0%) | 8 (0.5%) | 1 |
| Histology | | | 0.002 |
| Adenocarcinoma | 79 (97.5%) | 1269 (80.8%) | |
| Squamous cell carcinoma | 1 (1.2%) | 170 (10.8%) | |
| Small cell carcinoma | 1 (1.2%) | 79 (5.0%) | |
| Other | 0 | 52 (3.3%) | |
| Adenocarcinoma spectrum | | | <0.001 |
| AAH | 6 (7.4%) | 0 (0%) | |
| AIS | 7 (8.6%) | 0 (0%) | |
| MIA | 9 (11.1%) | 0 (0%) | |
| IPA | 57 (70.4%) | 1269 (100%) | |
| Stage | | | <0.001 |
| Carcinoma in situ | 14 (17.3%) | 4 (0.3%) | |
| I | 54 (66.7%) | 326 (20.8%) | |
| II | 4 (4.9%) | 64 (4.1%) | |
| III | 2 (2.5%) | 334 (21.3%) | |
| IV | 7 (8.6%) | 842 (53.6%) | |
| Curative surgery rate | 35 (83.3%) | 459 (29.2%) | <0.001 |
| Targeted therapy | 5 (11.9%) | 319 (20.3%) | 0.241 |
| Mean tumor size (mm) | 16.16 ± 13.74 | 41.25 ± 23.36 | <0.001 |
| Deaths | 8 (9.9%) | 1159 (73.8%) | <0.001 |

| | | | |
|---|---|---|---|
| Mean survival days | 892.05 ± 516.24 | 676.03 ± 600.47 | <0.001 |
| Median survival days | 825 (30-2599) | 517.5 (1-3128) | |
| Abbreviations: AAH: atypical adenomatous hyperplasia; AIS: adenocarcinoma in situ; IPA: invasive pulmonary adenocarcinoma; MIA: minimally invasive adenocarcinoma; SD: standard deviation. | | | |

**Supplement Table S2.** Clinical characteristics of 1232 smokers with lung cancer according to screening status.

| | **Screened (n = 12)** | **Non-Screened (n = 1220)** | ***p*-Value** |
|---|---|---|---|
| Mean age at diagnosis, years (mean, SD) | 63.33 ± 11.83 | 69.15 ± 13.00 | 0.118 |
| Median age at diagnosis, years (range) | 72 (42-83) | 71 (41-99) | |
| Gender (n, %) | | | 1.000 |
| Male | 12 (100%) | 1175 (96.3%) | |
| Female | 0 (0%) | 45 (3.7%) | |
| Smoking consumption | 12 (100%) | 1220 (100%) | |
| Alcohol drinking consumption | 6 (50%) | 405 (33.2%) | 0.230 |
| Betel nut | 3 (25%) | 187 (15.3%) | 0.411 |
| Histology | | | 0.210 |
| Adenocarcinoma | 9 (75%) | 744 (61%) | |
| Squamous cell carcinoma | 2 (16.7%) | 273 (22.4%) | |
| Small cell carcinoma | 0 (0%) | 178 (14.6%) | |
| Other | 1 (8.3%) | 25 (2%) | |
| Adenocarcinoma spectrum | | | |
| AAH | 0 (0%) | 0 (0%) | |
| AIS | 0 (0%) | 0 (0%) | |
| MIA | 0 (0%) | 0 (0%) | |
| IPA | 9 (75%) | 744 (60.9%) | |
| Stage | | | <0.001 |
| Carcinoma in situ | 0 (0%) | 0 (0%) | |
| I | 8 (66.7%) | 129 (10.6%) | |
| II | 1 (8.3%) | 60 (4.9%) | |

| | | | |
|---|---|---|---|
| III | 2 (16.7%) | 302 (24.8%) | |
| IV | 1 (8.3%) | 729 (59.8%) | |
| Curative surgery rate | 10 (83.3%) | 211 (17.3%) | <0.001 |
| Targeted therapy | 0 (0%) | 197 (16.1%) | 0.232 |
| Mean tumor size (mm) | 26.75 ± 19.99 | 51.12 ± 26.60 | 0.001 |
| Deaths | 2 (16.7%) | 1047 (85.8%) | <0.001 |
| Mean survival days | 646.08 ± 337.21 | 444.85 ± 468.43 | 0.064 |
| Median survival days | 683 (22-1217) | 304 (1-2937) | |
| Abbreviations: AAH: atypical adenomatous hyperplasia; AIS: adenocarcinoma in situ; IPA: invasive pulmonary adenocarcinoma; MIA: minimally invasive adenocarcinoma; SD: standard deviation. | | | |
